# Quantum Privacy-Preserving Range Query Protocol for Encrypted Data in IoT Environments

**DOI:** 10.3390/s24227405

**Published:** 2024-11-20

**Authors:** Chong-Qiang Ye, Jian Li, Xiao-Yu Chen

**Affiliations:** 1 School of Information and Electrical Engineering, Hangzhou City University, Hangzhou 310015, China; 2 School of Cyberspace Security, Beijing University of Posts and Telecommunications, Beijing 100876, China

**Keywords:** quantum communication, range query, privacy protection, IoT security

## Abstract

With the rapid development of IoT technology, securely querying sensitive data collected by devices within a specific range has become a focal concern for users. This paper proposes a privacy-preserving range query scheme based on quantum encryption, along with circuit simulations and performance analysis. We first propose a quantum private set similarity comparison protocol and then construct a privacy-preserving range query scheme for IoT environments. By leveraging the properties of quantum homomorphic encryption, the proposed scheme enables encrypted data comparisons, effectively preventing the leakage of sensitive data. The correctness and security analysis demonstrates that the designed protocol guarantees users receive the correct query results while resisting both external and internal attacks. Moreover, the protocol requires only simple quantum states and operations, and does not require users to bear the cost of complex quantum resources, making it feasible under current technological conditions.

## 1. Introduction

The Internet of Things (IoT) is a cutting-edge technology [1] that seamlessly integrates physical devices into the digital world, creating a network capable of real-time monitoring, control, and automation. It has profoundly reshaped interactions with technology and the management of resources and is continually impacting our daily lives.

IoT systems or environments typically have the following characteristics: (1) a large number of devices: IoT systems consist of a large number of interconnected devices, ranging from simple sensors to complex processing units; (2) a dynamic and distributed nature: IoT environments are highly dynamic, with devices constantly joining, leaving, or moving within the network; (3) security and privacy: Security and privacy protection are critical issues in IoT environments. Since IoT devices often collect large amounts of sensitive data (such as personal health information, location data, etc.), special attention is required for the secure storage, transmission, and processing of such data.

Ensuring data security while enabling meaningful analysis is a critical issue in IoT ecosystems [2]. As a result, there is a growing need for privacy-preserving data processing techniques that can perform complex queries on sensitive data without compromising individual privacy. Among the various types of queries that are central to IoT applications, range queries, which retrieve data that fall within a specific range, are particularly important [3,4]. For example, in smart city management, range queries can be used for environmental monitoring, e.g., to query air quality sensor data in a certain area, or to query temperature changes in multiple areas over a specific time period. These data can help city managers optimize resource allocation and emergency responses. However, executing range queries on unencrypted data can lead to the exposure of sensitive information, making privacy protection a critical concern.

To address these challenges, privacy-preserving range query techniques have emerged as a powerful solution [5,6,7,8]. In these privacy-preserving scenarios, the querying user does not reveal the specific range of the query, and the data owner does not disclose any individual element to others, except for the queried data. To illustrate the concept of privacy-preserving range queries, let us consider a typical scheme described in Ref. [8]. In this protocol, there are *m* data owners (e.g., IoT devices) and one query user. Each data owner $D_{i}$ has privacy data: $w_{i}$ within the range [1,n]. The query user wants to know “What is the sum of the elements with indices in the range/interval [l,u]? where 1≤l<u≤n”. In this specific example, the query user’s range, [l,u], is kept confidential, and the data owner’s $w_{i}$ will not be disclosed to others. Moreover, in addition to sum queries within a privacy-preserving range, queries for the maximum or minimum value are also frequently required in IoT applications. S. Sciancalepore et al. [9] and M. Zhou et al. [10] implemented privacy-preserving range query protocols for finding maximum or minimum values in IoT systems. These protocols utilize classical encryption techniques, such as homomorphic encryption [11], privacy comparison, and other algorithms.

However, traditional cryptographic protocols, which are based on mathematically hard problems, are increasingly vulnerable to the power of quantum computing. The emergence of quantum algorithms, such as Shor’s algorithm [12], poses a significant threat to the security of classical encryption schemes, indirectly compromising the data security of the IoT ecosystem. In contrast, quantum cryptographic protocols [13,14,15] are inherently resistant to quantum attacks, providing long-term security assurances even in the presence of quantum adversaries. In 2023, based on quantum multiparty computing XOR and quantum privacy query, Shi et al. [16] designed a quantum-based privacy-preserving range query scheme. By integrating quantum cryptography with classical IoT systems, this method significantly enhances the security of traditional range query applications. In 2024, Shi et al. [17] proposed a quantum-based privacy-preserving range MAX/MIN query scheme, leveraging quantum private query and quantum oblivious set inclusion protocols. Their approach effectively ensures the privacy of both the query range and the query results. In summary, classical encryption-based privacy-preserving range query protocols may be easier to implement in the short term, but their security could be compromised as quantum computing advances, offering only short-term security. On the other hand, quantum encryption-based solutions, which leverage the principles of quantum encryption, offer long-term security by resisting attacks from quantum computers. However, it is important to note that research on quantum solutions is still in its early stages. Integrating quantum encryption technologies into IoT systems requires further investigation, and its implementation faces higher costs and challenges compared to classical solutions.

### 1.1. Motivation

The primary motivations for proposing the scheme in this paper are as follows:

- Given the rapidly increasing computational power, quantum cryptographic protocols have the potential to become a preferred solution for safeguarding the vast amounts of sensitive data generated by IoT devices. To the best of our knowledge, only Shi et al. [16,17] have proposed two relevant schemes suitable for IoT environments. This is clearly insufficient to meet the growing security demands. *Thus, there is a clear need to explore further integration of quantum encryption technologies within IoT systems.*
- Quantum homomorphic encryption (QHE) [18,19] is an advanced encryption technology that combines the principles of quantum computing with homomorphic encryption. Similar to classical homomorphic encryption, QHE allows computations to be performed directly on encrypted data without the need for decryption. In classical privacy-preserving range queries, homomorphic encryption is a key component. *However, there is currently no solution that employs quantum homomorphic encryption to address privacy-preserving range queries.*

These motivation inspired us to design a quantum-based privacy-preserving range query protocol for IoT systems.

### 1.2. Research Contributions

In this paper, we present a quantum privacy-preserving range query protocol for IoT environments. The design of the proposed scheme was inspired by the research findings of [17,20]. With the help of a semi-honest quantum cloud server, users can perform range queries on the data owners, such as edge servers. The proposed protocol employs QHE, ensuring that the quantum cloud server processes only encrypted data, thereby safeguarding sensitive information within the IoT system. The main contributions are summarized as follows.

(1) We propose a privacy set similarity comparison protocol based on quantum homomorphic encryption, which allows for the comparison of encrypted data.
(2) Based on the proposed privacy set similarity comparison protocol, we further give a feasible quantum privacy-preserving range query protocol for IoT environments.
(3) The quantum circuits corresponding to the proposed protocol are presented, and their feasibility is validated through simulation.

### 1.3. Organization

The rest of this paper is organized as follows. Section 2 briefly overviews the quantum resources utilized in this approach. Section 3 outlines the detailed steps of the proposed protocol. In Section 4, we discuss the protocol’s correctness and present circuit simulation results. Section 5 covers the security analysis. Finally, the discussion and conclusions are presented in Section 6 and Section 7.

## 2. Preliminaries

In this section, we provide an overview of the quantum resources to be used in this paper, including basic quantum gates and quantum homomorphic encryption.

### 2.1. Basic Quantum Gates

In quantum systems, quantum gates are the fundamental building blocks used to manipulate qubits. Below is an introduction to some of the basic quantum gates.

(1) Pauli Gate (X, Y, Z Gate): Pauli gates form a set of fundamental single-qubit gates that correspond to classical bit flips and phase flips.
  X Gate (bit-flip gate): Similar to the classical NOT gate, it flips |0〉 to |1〉 and vice versa.
  Y Gate: It both performs a bit flip and introduces a phase change. It maps |0〉 to i|1〉 and |1〉 to −i|0〉.
  Z Gate (phase-flip gate): This gate only changes the phase of the qubit. It multiplies the |1〉 state by −1, while leaving |0〉 unchanged.
(2) Hadamard Gate (H Gate): The Hadamard gate is one of the most commonly used single-qubit gates. It maps |0〉 to $(|0\rangle +\left|1\rangle \right)/\sqrt{2}$, |1〉 to $(|0\rangle -\left|1\rangle \right)/\sqrt{2}$ and vice versa.
(3) CNOT Gate (Controlled-NOT Gate): The CNOT gate is a two-qubit gate where one qubit acts as the control and the other as the target. If the control qubit is |1〉, the target qubit undergoes an X gate operation (bit flip); if the control qubit is |0〉, the target qubit remains unchanged.

### 2.2. Quantum Homomorphic Encryption

Quantum homomorphic encryption (QHE) is a cryptographic technique that allows computations to be performed directly on encrypted quantum data, without the need for decryption [18,19]. This capability is essential in privacy-preserving quantum computing, where a client wishes to delegate computations to a quantum server while maintaining the confidentiality of the underlying data.

The main advantage of QHE is that the server never learns anything about the input quantum data, intermediate states, or the final result, thus ensuring the confidentiality of the computation. Generally, a QHE scheme involves four stages:

- **Key Generation.** *QHE.KeyGen*: $1^{\kappa }$→($pk,sk,\rho_{evk}$). This process takes the unary representation of the security parameter as input and generates the classical keys pk and sk, along with a quantum evaluation key $\rho_{evk}$ as output.
- **Encryption.** *QHE.Enc_pk_*: D(M)→D(C). This process uses the key pk to transform the message space M into the cipherspace C.
- **Evaluation.** *QHE*.${\mathrm{Eval}}_{\rho_{evk}}^{QC}$: $D\left(C\right)\to D\left(C^{\prime }\right)$. Based on the evaluation key $\rho_{evk}$, a quantum evaluation circuit QC is applied to the ciphertext C, and then it produces a new quantum ciphertext state $C^{\prime }$.
- **Decryption.** *QHE.Dec_sk_*: $D(C^{\prime })\to \rho$. Using the private key sk, the ciphertext $C^{\prime }$ is decrypted to recover the plaintext state ρ, where ρ represents the output of the quantum evaluation circuit applied to the original plaintext D(M).

QHE can be categorized into two main types: limited and fully homomorphic encryption. Limited QHE supports a restricted set of quantum operations, usually within the Clifford group, which can be easily implemented on encrypted data. Fully quantum homomorphic encryption (FQHE), on the other hand, supports arbitrary quantum operations, but it is still challenging to realize practical FQHE, due to the difficulty of implementing non-Clifford gates such as the *T* gate in an encrypted form. This paper focuses on the quantum homomorphic encryption of the CNOT gate, which is a member of the Clifford group.

The homomorphic encryption process for the CNOT gate can be summarized as follows: First, the quantum encryption key is established through a key generation process, where parameters α and β ($\alpha ,\beta \in {\left\{0,1\right\}}^{n}$) belong to the key pk. Next, the quantum states $|\phi_{k}\rangle$ and $|\psi_{l}\rangle$ are encrypted using $X^{\alpha_{k}}Z^{\beta_{k}}$ and $X^{\alpha_{l}}Z^{\beta_{l}}$, respectively, resulting in encrypted quantum states that ensure confidentiality during transmission. The encrypted quantum states $X^{\alpha_{k}}Z^{\beta_{k}}|\phi_{k}\rangle$ and $X^{\alpha_{l}}Z^{\beta_{l}}|\psi_{l}\rangle$ are then sent to the quantum server, where the server performs the homomorphic evaluation of the CNOT gate directly on the encrypted states, ensuring the privacy of the data. Finally, the decryption operation is performed using the key sk (i.e., $\alpha_{k},\beta_{k}⊕\beta_{l},\alpha_{k}⊕\alpha_{l},\beta_{l}$), and the corresponding quantum state is measured to obtain the final result. The whole process is also shown in Equation (1):

(1) $$\begin{array}{cc} \; & \overset{\mathrm{key}\;\mathrm{generation}\;}{\to }\left(\alpha_{k},\beta_{k}\right),\left(\alpha_{l},\beta_{l}\right)\\ \; & \overset{\mathrm{encryption}\;}{\to }\left(X^{\alpha_{k}}Z^{\beta_{k}}⊗X^{\alpha_{l}}Z^{\beta_{l}}\right)|\phi_{k}\rangle |\psi_{l}\rangle \\ \; & \overset{\mathrm{CNOT}\;}{\to }\left(X^{\alpha_{k}}Z^{\beta_{k}⊕\beta_{l}}⊗X^{\alpha_{k}⊕\alpha_{l}}Z^{\beta_{l}}\right)|\phi_{k}\rangle |\phi_{k}⊕\psi_{l}\rangle \\ \; & \overset{\mathrm{decryption}\;}{\to }|\phi_{k}\rangle ,|\phi_{k}⊕\psi_{l}\rangle \end{array}.$$

## 3. Protocol Description

In this section, we first give a QHE-based privacy set similarity comparison scheme. Then, a privacy-preserving range query scheme is provided based on the designed privacy set comparison method.

### 3.1. Quantum Privacy Set Similarity Comparison Protocol Based on QHE

Before initiating the protocol, let us first introduce the basic requirements of the protocol. This protocol involves three participants: the server, Alice; the client, Bob; and a quantum cloud third party (TP). Alice and Bob each possess a private set, denoted as $S_{A}=\left\{x_{1},x_{2},\cdots ,x_{n}\right\}\subseteq Z_{N}$ and $S_{B}=\left\{y_{1},y_{2},\cdots ,y_{m}\right\}\subseteq Z_{N}$, respectively. With the assistance of TP, they aim to compare their private sets’ similarity using quantum homomorphic encryption based on CNOT gates.

In this paper, we utilize the Jaccard similarity [21] to measure the similarity between the two sets, which is defined as

(2) $$\begin{array}{c} J=\frac{|S_{A}\cap S_{B}|}{|S_{A}\cup S_{B}|}. \end{array}$$

The detailed protocol step is described below.

***1. Privacy set encoding phase:*** Alice and Bob encode their respective private datasets, transforming them into quantum state sequences.

Step 1-1: Alice and Bob execute a quantum key distribution protocol [22] to establish an integer key $k\subseteq Z_{N}$. Then, the sets of $S_{A}$ and $S_{B}$ are transformed into specific privacy vectors: $S_{A}*=\left\{kx_{1}\;\mathrm{mod}\;N,kx_{2}\;\mathrm{mod}\;N,\cdots ,kx_{n}\;\mathrm{mod}\;N\right\}$ and $S_{B}*=\left\{ky_{1}\;\mathrm{mod}\;N,ky_{2}\;\mathrm{mod}\;N,\cdots ,ky_{m}\;\mathrm{mod}\;N\right\}$. The transformation above merely applies a uniform modular multiplication to the elements of sets $S_{A}$ and $S_{B}$, without altering the intersection or union relationships between the two sets or the size of the set.

Step 1-2: using the sets $S_{A}*$ and $S_{B}*$, Alice and Bob generate respective quantum sequences $(|A_{0}\rangle ,|A_{1}\rangle ,\cdots ,|A_{N-1}\rangle )$ and $(|B_{0}\rangle ,|B_{1}\rangle ,\cdots ,|B_{N-1}\rangle )$ in the following manner:

(3) $$\begin{array}{ccc} \; & |A_{i}\rangle =|0\rangle ,\;\mathrm{if}\;i\notin S_{A}*,\; & |A_{i}\rangle =|1\rangle ,\;\mathrm{if}\;i\in S_{A}*,\\ \; & |B_{i}\rangle =|0\rangle ,\;\mathrm{if}\;i\notin S_{B}*,\; & |B_{i}\rangle =|1\rangle ,\;\mathrm{if}\;i\in S_{B}*, \end{array}$$

where i=0,1,⋯,N−1. It should be noted that after the encoding phase, the number of |1〉 in the quantum state sequences prepared by each Alice and Bob is *n* and *m*, respectively, which is consistent with the original set size.

***2. Key generation phase:*** TP, Alice, and Bob execute the quantum secret sharing protocol to establish the key relationship.

Step 2-1: TP first prepares 8N+δ Bell states, with each Bell state randomly belonging to either the $|\Phi \rangle =\frac{1}{\sqrt{2}}(|00\rangle +\left|11\rangle \right)$ or $|\Psi \rangle =\frac{1}{\sqrt{2}}(|01\rangle +\left|10\rangle \right)$. TP then uses $R_{j}^{T}$ to record the different types of Bell states prepared. If the *j*-th Bell state is |Φ〉, then $R_{j}^{T}=0$; otherwise, if it is a |Ψ〉 state, $R_{j}^{T}=1$. After that, TP splits these Bell states into two particle sequences, $T_{A}$ and $T_{B}$, which are subsequently sent to Alice and Bob.

Step 2-2: For the received qubits, Alice and Bob randomly perform Z-basis (i.e., {|0〉,|1〉}) measurements or X-basis (i.e., $\{|+\rangle =\frac{1}{\sqrt{2}}(|0\rangle +|1\rangle ),|-\rangle =\frac{1}{\sqrt{2}}(|0\rangle -\left|1\rangle )\right\}$) measurements.

Step 2-3: Alice and Bob randomly select δ qubits as test qubits to perform a security check. They first instruct TP to reveal the type of Bell state for these test particles, namely whether they are in the |Φ〉 or |Ψ〉 state. Based on the measurement basis and the corresponding outcomes, Alice and Bob can assess the security of the transmission and verify whether TP has prepared the Bell states as required by the protocol. The focus here is on the case where Alice and Bob choose the same measurement basis. The relationship between the measurement basis and the results is summarized in Table 1.

If the error rate of any of the eight cases in Table 1 exceeds the threshold, the protocol will terminate and restart. Otherwise, proceed to the next step.

Step 2-4: After confirming the security of the channel and verifying that TP has correctly prepared the Bell states as required, Alice and Bob select the particles on which they both performed Z-basis measurements (e.g., the two particles in the *j*-th Bell state). The corresponding measurement results are recorded as $R_{j}^{A}$ and $R_{j}^{B}$, respectively. Correspondingly, Alice and Bob will also tell TP the position of the selected particle.

Step 2-5: TP, Alice, and Bob can establish a key relationship based on $R_{j}^{T}$, $R_{j}^{A}$, and $R_{j}^{B}$. Based on the properties of the Bell states |Ψ〉 and |Φ〉, it can be easily derived that

(4) $$R_{j}^{T}=R_{j}^{A}⊕R_{j}^{B},$$

where $R_{j}^{T}$ is the Bell state type recorded in Step 2-1 (also see cases 1, 2, 5, and 6 of Table 1).

It is important to highlight that in this phase, TP only has access to the XOR result of $R_{j}^{A}$ and $R_{j}^{B}$, without the ability to retrieve the individual values of $R_{j}^{A}$ or $R_{j}^{B}$ independently.

***3. Quantum homomorphic encryption phase:*** Alice and Bob utilize the keys generated in the key generation phase, labeled here as $R_{i}^{A}$ and $R_{i}^{B}$, to encrypt their individual quantum states. They then send the encrypted states to TP for the execution of the CNOT homomorphic evaluation and decryption.

Step 3-1: Alice and Bob use $R_{i}^{A}=\left(\alpha_{i}^{A},\beta_{i}^{A}\right)$ and $R_{i}^{B}=\left(\alpha_{i}^{B},\beta_{i}^{B}\right)$ to encrypt the *i*-th states $|A_{i}\rangle$ and $|B_{i}\rangle$. The encrypted quantum states are labeled as $X^{\alpha_{i}^{A}}Z^{\beta_{i}^{A}}|A_{i}\rangle$ and $X^{\alpha_{i}^{B}}Z^{\beta_{i}^{B}}|B_{i}\rangle$, respectively.

Step 3-2: Alice and Bob then send the encrypted quantum states to TP for further CNOT homomorphic evaluation.

Step 3-3: TP performs the CNOT-gate on the *i*-th states $X^{\alpha_{i}^{A}}Z^{\beta_{i}^{A}}|A_{i}\rangle$ and $X^{\alpha_{i}^{B}}Z^{\beta_{i}^{B}}|B_{i}\rangle$, yielding

(5) $$\begin{array}{cc} \; & CNOT\left(X^{\alpha_{i}^{A}}Z^{\beta_{i}^{A}}⊗X^{\alpha_{i}^{B}}Z^{\beta_{i}^{B}}\right)|A_{i}\rangle |B_{i}\rangle \\ \; & \;=\left(X^{\alpha_{i}^{A}}Z^{\beta_{i}^{A}⊕\beta_{i}^{B}}⊗X^{\alpha_{i}^{A}⊕\alpha_{i}^{B}}Z^{\beta_{i}^{B}}\right)|A_{i}\rangle |A_{i}⊕B_{i}\rangle \end{array}.$$

According to Equation (4), i.e., $R_{i}^{T}=R_{i}^{A}⊕R_{i}^{B}$, TP can derive the value of $\alpha_{i}^{A}⊕\alpha_{i}^{B}$ based on $R_{i}^{T}$. Subsequently, TP performs the decryption operation $X^{\alpha_{i}^{A}⊕\alpha_{i}^{B}}$ on the second particle, corresponding to the second half of Equation (5). Specifically, this operation is carried out as shown in Equation (6).

(6) $$\begin{array}{cc} \; & X^{\alpha_{i}^{A}}Z^{\beta_{i}^{A}⊕\beta_{i}^{B}}|A_{i}\rangle ⊗X^{\alpha_{i}^{A}⊕\alpha_{i}^{B}}X^{\alpha_{i}^{A}⊕\alpha_{i}^{B}}Z^{\beta_{i}^{B}}|A_{i}⊕B_{i}\rangle \\ \; & \;=X^{\alpha_{i}^{A}}Z^{\beta_{i}^{A}⊕\beta_{i}^{B}}|A_{i}\rangle ⊗Z^{\beta_{i}^{B}}|A_{i}⊕B_{i}\rangle \end{array}.$$

Since both $|A_{i}\rangle$ and $|B_{i}\rangle$ belong to {|0〉,|1〉}, the CNOT operation does not cause entanglement of $|A_{i}\rangle$ and $|B_{i}\rangle$, so the final quantum states $|A_{i}\rangle$ and $|A_{i}⊕B_{i}\rangle$ are independent.

Step 3-4: TP performs the Z-basis measurements on the particle $|A_{i}⊕B_{i}\rangle$ to obtain the comparison results. Note that the application of the Z-gate does not cause a state flip but only induces a phase shift. As a result, during Z-basis measurements, this phase shift has no effect, and thus, the influence of the Z-gate on the final outcome is disregarded.

Based on the measurement results, TP can infer the relationship between Alice’s and Bob’s private sets (see Table 2). Specifically, if $|A_{i}⊕B_{i}\rangle =|0\rangle$, it indicates that element *i* either belongs to $S_{A}*\cap S_{B}*$ or ${\left(S_{A}*\cup S_{B}*\right)}^{c}$. Here, ${\left(S_{A}*\cup S_{B}*\right)}^{c}$ denotes the complement of the union of sets $S_{A}*$ and $S_{B}*$, meaning all elements that are in neither $S_{A}*$ nor $S_{B}*$. Conversely, if $|A_{i}⊕B_{i}\rangle =|1\rangle$, it signifies that element *i* belongs to the symmetric difference of $S_{A}*$ and $S_{B}*$, i.e., $S_{A}*\Delta S_{B}*=\left(S_{A}*-S_{B}*\right)\cup \left(S_{B}*-S_{A}*\right)$.

***4. Set similarity calculation phase:*** Alice and Bob inform TP of the sizes of their respective sets, i.e., $|S_{A}\left|=\right|S_{A}*|=n$ and $|S_{B}\left|=\right|S_{B}*|=m$. Based on the measurement results, TP computes the set similarity and announces the outcome to Alice and Bob.

Step 4-1: Alice and Bob send their respective set sizes *n* and *m* to TP through a quantum secure direct communication protocol [23].

Step 4-2: TP counts the size of $|S_{A}*\Delta S_{B}*|$, i.e., the number of measurements with the result of |1〉 in Step 3-4, labeled as *l*. TP then performs the following calculations to derive the intersection and union sizes of sets $S_{A}*$ and $S_{B}*$:

(7) $$\begin{array}{cc} \; & |S_{A}*\cap S_{B}*|=(|S_{A}*|+|S_{B}*|-|S_{A}*\Delta S_{B}*\left|\right)/2=\left(n+m-l\right)/2\\ \; & |S_{A}*\cup S_{B}*|=|S_{A}*|+|S_{B}*|-|S_{A}*\cap S_{B}*|=n+m-\left(n+m-l\right)/2 \end{array}.$$

Thereby, TP can obtain the set similarity of $S_{A}*$ and $S_{B}*$ (i.e., $S_{A}$ and $S_{B}$).

(8) $$\begin{array}{c} J_{AB}=\frac{|S_{A}*\cap S_{B}*|}{|S_{A}*\cup S_{B}*|}=\frac{|S_{A}\cap S_{B}|}{|S_{A}\cup S_{B}|}=\frac{n+m-l}{n+m+l} \end{array}.$$

Finally, TP publishes the set similarity results to Alice and Bob.

### 3.2. Quantum Privacy-Preserving Range Query Protocol

**Figure 1:**
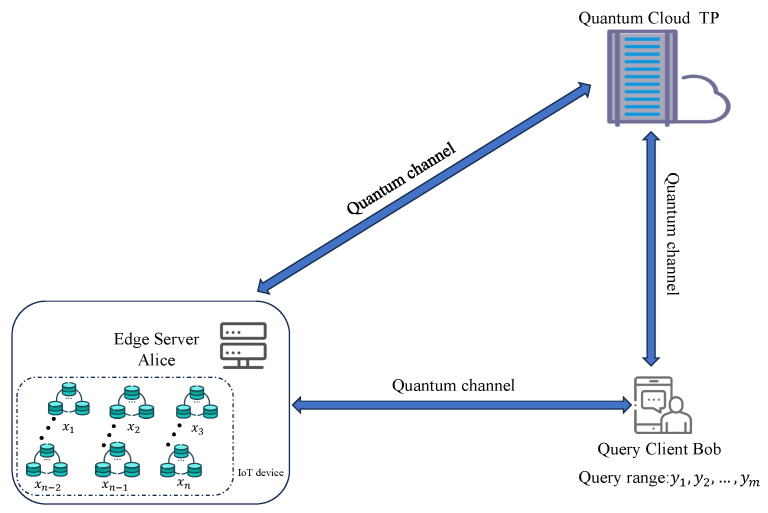
Basic protocol model.

In this part, a privacy-preserving range query protocol in the IoT environments is proposed, where the protocol model is depicted in Figure 1.

This model involves three participants: Alice, Bob, and TP. Specifically, Alice acts as an edge server responsible for managing sensitive data collected from multiple IoT devices. The complete dataset can be represented as follows:

(9) $$\begin{array}{cc} \; & x_{1}:\left\{d_{1}^{1},d_{1}^{2},\cdots ,d_{1}^{G}\right\}\\ \; & x_{2}:\left\{d_{2}^{1},d_{2}^{2},\cdots ,d_{2}^{G}\right\}\\ \; & \;\vdots \;\;\vdots \\ \; & x_{n}:\left\{d_{n}^{1},d_{n}^{2},\cdots ,d_{n}^{G}\right\} \end{array},$$

where $x_{h}$ (h=1,2,⋯,n) denotes the *h*-th IoT device’s index number, and $\left\{d_{h}^{1},d_{h}^{2},\cdots ,d_{h}^{G}\right\}$ denotes the collected data of the *h*-th IoT device, with each $d_{h}^{g}\in \left[0,N\right)$ (g=1,2,⋯,G). Here, the index numbers $x_{1},x_{2},\cdots ,x_{n}$ constitute a privacy set, denoted as SA.

Bob is a querying user who wishes to privately retrieve data from a specific range of IoT device nodes managed by Alice. Bob’s query range is denoted as $y_{1},y_{2},\cdots ,y_{m}$, which also forms a set, denoted as SB. TP is a semi-honest quantum cloud to assist with the privacy query task. However, TP is considered curious and may attempt to infer both Alice’s data and the result of Bob’s query.

The goal of this protocol is to enable privacy-preserving range queries between the edge server, Alice, and the querying client, Bob, with the assistance of the quantum cloud, TP, using quantum encryption techniques. The protocol’s security requirements are twofold: (1) to ensure the privacy of both Bob’s query range and the corresponding query results, and (2) to protect the privacy of all data elements in Alice’s dataset that are not part of the query result. The detailed steps are as follows.

Step 1: Alice and Bob execute the first phase of the protocol in Section 3.1 to transform the two privacy sets SA and SB into SA* and SB*.

(10) $$\begin{array}{cc} \; & SA*=\left\{x_{1}^{\prime },x_{2}^{\prime },\cdots ,x_{n}^{\prime }\right\},\;SB*=\left\{y_{1}^{\prime },y_{2}^{\prime },\cdots ,y_{m}^{\prime }\right\}, \end{array}$$

where $x_{h}^{\prime }=kx_{h}\;\mathrm{mod}\;N$ and $y_{w}^{\prime }=ky_{w}\;\mathrm{mod}\;N$ (w=1,2⋯,m). Accordingly, Alice’s original dataset is modified to be

(11) $$\begin{array}{cc} \; & x_{1}^{\prime }:\left\{d_{1}^{1},d_{1}^{2},\cdots ,d_{1}^{G}\right\}\\ \; & x_{2}^{\prime }:\left\{d_{2}^{1},d_{2}^{2},\cdots ,d_{2}^{G}\right\}\\ \; & \;\vdots \;\;\vdots \\ \; & x_{n}^{\prime }:\left\{d_{n}^{1},d_{n}^{2},\cdots ,d_{n}^{G}\right\} \end{array},$$

Then, the two quantum sequences $(|A_{0}\rangle ,|A_{1}\rangle ,\cdots ,|A_{N-1}\rangle )$ and $(|B_{0}\rangle ,|B_{1}\rangle ,\cdots ,|B_{N-1}\rangle )$ are modified in the following manner:

(12) $$\begin{array}{ccc} \; & |A_{i}\rangle =|0\rangle ,\;\mathrm{if}\;i\neq x_{h}^{\prime },\; & |A_{i}\rangle =|1\rangle ,\;\mathrm{if}\;i=x_{h}^{\prime },\\ \; & |B_{i}\rangle =|0\rangle ,\;\mathrm{if}\;i\neq y_{w}^{\prime },\; & |B_{i}\rangle =|1\rangle ,\;\mathrm{if}\;i=y_{w}^{\prime }, \end{array}$$

Step 2: Alice and Bob establish *N* integer keys $K_{AB}=\left\{k_{0},k_{1}\cdots ,k_{N-1}\right\}$ via the QKD protocol. Similarly, Alice and TP, as well as Bob and TP, each establish *N* integer keys $K_{AT}=\left\{t_{0},t_{1},\cdots ,t_{N-1}\right\}$ and $K_{BT}=\left\{b_{0},b_{1},\cdots ,b_{N-1}\right\}$, respectively.

Step 3: According to the state of $|A_{i}\rangle$, Alice applies different manners to encrypt the private data. If $|A_{i}\rangle =|1\rangle$, it means that *i* belongs to the set SA*, i.e., $i=x_{h}^{\prime }$; then, the corresponding privacy data $\left\{d_{h}^{1},d_{h}^{2},\cdots ,d_{h}^{G}\right\}$ will be transformed to

(13) $$\begin{array}{c} d_{h}^{1}+k_{i}+t_{i},\;d_{h}^{2}+k_{i}+t_{i},\;\cdots ,\;d_{h}^{G}+k_{i}+t_{i} \end{array}.$$

If $|A_{i}\rangle =|0\rangle$, it indicates that *i* is not part of the set SA*, meaning $i\neq x_{h}^{\prime }$. At this position, there were no original data, so Alice must generate a new set of identical data for padding. In this case, the generated data are assumed to be

(14) $$\begin{array}{c} a_{i}+k_{i}+t_{i},\;a_{i}+k_{i}+t_{i},\;\cdots ,\;a_{i}+k_{i}+t_{i} \end{array}.$$

where $a_{i}$ is random and only known to Alice. Thus, after encryption, Alice’s private dataset can be expressed as

(15) $$\left\{\begin{array}{c} x_{h}^{\prime }:\left\{d_{h}^{1}+k_{i}+t_{i},\;d_{h}^{2}+k_{i}+t_{i},\;\cdots ,\;d_{h}^{G}+k_{i}+t_{i}\right\},\;if\;|A_{i}\rangle =|1\rangle ,i.e.,i=x_{h}^{\prime }\\ a_{i}^{\prime }:\left\{a_{i}+k_{i}+t_{i},\;a_{i}+k_{i}+t_{i},\;\cdots ,\;a_{i}+k_{i}+t_{i}\right\},\;if\;|A_{i}\rangle =|0\rangle ,i.e.,i\neq x_{h}^{\prime } \end{array}\right.,$$

where $a_{i}^{\prime }$ corresponds to the position where $|A_{i}\rangle =|0\rangle$, and the private data generated at these positions are the same.

Step 4: Executing the key generation phase of the protocol in Section 3.1, Alice, Bob and TP establish a key relationship as shown in Equation (4).

Step 5: Using the established keys, Alice and Bob encrypt their respective quantum state sequences, $(|A_{0}\rangle ,|A_{1}\rangle ,\cdots ,|A_{N-1}\rangle )$ and $(|B_{0}\rangle ,|B_{1}\rangle ,\cdots ,|B_{N-1}\rangle )$, before sending them to TP for quantum homomorphic evaluation. This corresponds to executing the quantum homomorphic encryption phase of the protocol in Section 3.1.

Step 6: According to Step 3-4 listed in Section 3.1, TP can obtain the value of $|A_{i}⊕B_{i}\rangle$ through the Z-basis measurement. The focus here is on cases where the measurement result is |0〉. If $|A_{i}⊕B_{i}\rangle =|0\rangle$, TP records the corresponding value of *i*. Then, based on the value of *i* and the keys $t_{i}$ and $b_{i}$, TP generates a privacy vector $V=[v_{0},v_{1},\cdots ,v_{N-1}]$ as follows:

(16) $$v_{i}=\left\{\begin{array}{cc} t_{i}-b_{i},\; & \mathrm{if}\;|A_{i}⊕B_{i}\rangle =|0\rangle \\ 0,\; & \mathrm{if}\;|A_{i}⊕B_{i}\rangle =|1\rangle \end{array}\right..$$

Step 7: Through a quantum secure direct communication protocol, Alice sends the privacy data corresponding to Equation (15) to TP. There are two situations to consider here. First, consider the case where $|A_{i}\rangle =|1\rangle$, i.e., $i=x_{h}^{\prime }$. In this case, Alice sends the data as $\left\{d_{h}^{1}+k_{i}+t_{i},d_{h}^{2}+k_{i}+t_{i},\cdots ,d_{h}^{G}+k_{i}+t_{i}\right\}$. In contrast, when $|A_{i}\rangle =|0\rangle$, Alice sends the data as $\left\{a_{i}+k_{i}+t_{i},a_{i}+k_{i}+t_{i},\cdots ,a_{i}+k_{i}+t_{i}\right\}$. TP can easily distinguish whether $|A_{i}\rangle$ equals |0〉 or |1〉 based on the data sent by Alice. This is because when $|A_{i}\rangle =|0\rangle$, the data Alice sends are identical across all entries.

For the *i*-th set of data, where $|A_{i}⊕B_{i}\rangle =|0\rangle$ and $|A_{i}\rangle =|1\rangle$, TP decrypts using the corresponding $v_{i}$ to retrieve a new set of private data. Specifically, TP subtracts $v_{i}$ from the data sent by Alice to obtain the final result:

(17) $$\begin{array}{c} \{d_{h}^{1}+k_{i}+b_{i},\;d_{h}^{2}+k_{i}+b_{i},\;\cdots ,\;d_{h}^{G}+k_{i}+b_{i}\}, \end{array}$$

where position *i* satisfies $|A_{i}⊕B_{i}\rangle =|0\rangle$ and $|A_{i}\rangle =|1\rangle$.

For position *i* that does not meet the above conditions, TP prepares a set of identical random data as padding, denoted as $\left\{p_{i}^{1},p_{i}^{2},\cdots ,p_{i}^{G}\right\}$.

Step 8: Through a quantum secure direct communication protocol, TP sends $\left\{d_{h}^{1}+k_{i}+b_{i},\;d_{h}^{2}+k_{i}+b_{i},\;\cdots ,\;d_{h}^{G}+k_{i}+b_{i}\right\}$ and $\left\{p_{i}^{1},p_{i}^{2},\cdots ,p_{i}^{G}\right\}$ to Bob. Here, Bob is only concerned with the case where $|B_{i}\rangle =|1\rangle$, because only these positions satisfy $|A_{i}\rangle =|B_{i}\rangle =|1\rangle$ and SA*∩SB*. For other positions *i*, Bob will only obtain the same result $\left\{p_{i}^{1},p_{i}^{2},\cdots ,p_{i}^{G}\right\}$.

Then, Bob uses the keys $k_{i}$ and $b_{i}$ to decrypt and retrieve the private data relevant to his query. Thus, for the *i*-th ($i=x_{h}^{\prime }=y_{w}^{\prime }$, i.e., $|A_{i}\rangle =|B_{i}\rangle =|1\rangle$) group of data $\left\{d_{h}^{1}+k_{i}+b_{i},\;d_{h}^{2}+k_{i}+b_{i},\;\cdots ,\;d_{h}^{G}+k_{i}+b_{i}\right\}$, Bob performs the following calculation to obtain the final query results:

(18) $$\begin{array}{cc} \; & d_{h}^{1}+k_{i}+b_{i}-k_{i}-b_{i}=d_{h}^{1},\\ \; & d_{h}^{2}+k_{i}+b_{i}-k_{i}-b_{i}=d_{h}^{2},\\ \; & \;\vdots \\ \; & d_{h}^{G}+k_{i}+b_{i}-k_{i}-b_{i}=d_{h}^{G}. \end{array}$$

It is important to note that Bob can only access information within his queried range. For other cases, he cannot retrieve any data. This is because TP only reveals Alice’s data for indices *i* that belong to the intersection of sets SA* and SB*. For indices *i* in the other positions, Bob’s decryption result will uniformly be $\left\{p_{i}^{1},p_{i}^{2},\cdots ,p_{i}^{G}\right\}$, which contains no private information from Alice.

## 4. Correctness Analysis and Simulation

In this section, we will analyze the correctness of the protocol and conduct circuit simulations. We first analyze the correctness of the proposed quantum privacy set similarity comparison protocol, and then discuss the correctness of the privacy range query scheme applicable to IoT environments.

### 4.1. Correctness of the Proposed Quantum Privacy Set Similarity Comparison Protocol

Assuming that Alice and Bob’s privacy datasets are $S_{A}=\left\{2,3,5,6\right\}\subseteq Z_{7}$ and $S_{B}=\left\{1,2,5\right\}\subseteq Z_{7}$, respectively, the analysis proceeds step by step according to the four phases of the protocol.

In the privacy set encoding stage, we assume that Alice and Bob’s privacy sets are transformed into $S_{A}*=\left\{2\times 2\;\mathrm{mod}\;7,2\times 3\;\mathrm{mod}\;7,2\times 5\;\mathrm{mod}\;7,2\times 6\;\mathrm{mod}\;7\right\}=\left\{4,6,3,5\right\}$ and $S_{B}*=\left\{2\times 1\;\mathrm{mod}\;7,2\times 2\;\mathrm{mod}\;7,2\times 5\;\mathrm{mod}\;7\right\}=\left\{2,4,3\right\}$, where k=2. Thus, the quantum state sequences generated by Alice and Bob are, respectively,

(19) $$\begin{array}{cc} \; & |A_{0}\rangle =|0\rangle ,|A_{1}\rangle =|0\rangle ,|A_{2}\rangle =|0\rangle ,|A_{3}\rangle =|1\rangle ,\\ \; & |A_{4}\rangle =|1\rangle ,|A_{5}\rangle =|1\rangle ,|A_{6}\rangle =|1\rangle ,\\ \; & |B_{0}\rangle =|0\rangle ,|B_{1}\rangle =|0\rangle ,|B_{2}\rangle =|1\rangle ,|B_{3}\rangle =|1\rangle ,\\ \; & |B_{4}\rangle =|1\rangle ,|B_{5}\rangle =|0\rangle ,|B_{6}\rangle =|0\rangle . \end{array}$$

**Figure 2:**
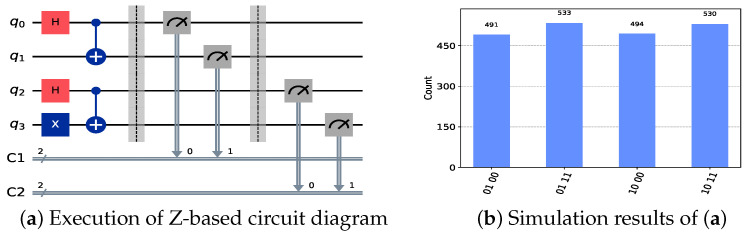
Circuits and simulation results for Z-basis measurements. Classical registers C1 and C2, respectively, record the Z-basis measurement results of |Φ〉 and |Ψ〉 by Alice and Bob. In subfigure (**b**), the horizontal axis represents the measurement results of the corresponding qubits, while the vertical axis indicates the frequency of each result.

**Figure 3:**
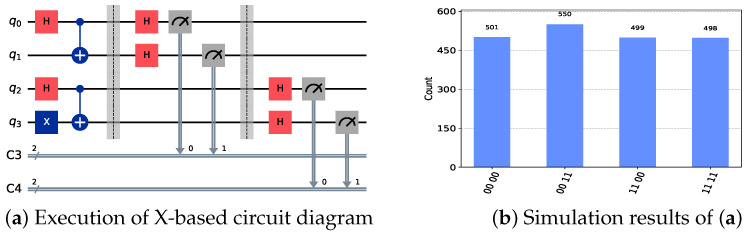
Circuits and simulation results for X-basis measurements. Classical registers C3 and C4, respectively, record the X-basis measurement results of |Φ〉 and |Ψ〉 by Alice and Bob. The horizontal axis in subfigure (**b**) represents the measurement results of the corresponding qubits, while the vertical axis shows the frequency of each result.

Then, in the key generation phase, Alice and Bob can establish a shared key with TP using Bell states, as described in Equation (4). Specifically, TP prepares and sends two sets of quantum states, |Ψ〉 and |Φ〉, to Alice and Bob, respectively. Both Alice and Bob then perform measurements using the Z-basis and X-basis. The corresponding quantum circuit simulation and results are shown in Figure 2 and Figure 3. It should be noted that in the simulation result diagrams of Figure 2 and Figure 3, the values on the horizontal axis represent the measurement results of the corresponding qubits, while the vertical axis represents the number of times the corresponding results appear. The number of simulations in the experiment is 2048.

The focus here is on the cases where Alice and Bob choose the same measurement basis. Only in these cases can the resulting particles be used for eavesdropping detection and key generation.

As seen in Figure 2, when both Alice and Bob choose Z-basis measurements, their results for quantum state |Φ〉 are identical, whereas for quantum state |Ψ〉, their measurement outcomes are opposite. This observation is consistent with the results in Table 1. When both Alice and Bob select X-basis measurements, their outcomes for both quantum states |Φ〉 and |Ψ〉 are the same, with the corresponding results shown in Figure 3. We assume here that the key relationship established between Alice, Bob, and TP is

(20) $$\begin{array}{cc} \; & R_{0}^{T}=\left(\alpha_{0}^{T}=0,\beta_{0}^{T}=1\right),\;R_{0}^{A}=\left(\alpha_{0}^{A}=1,\beta_{0}^{A}=1\right),\;R_{0}^{B}=\left(\alpha_{0}^{B}=1,\beta_{0}^{B}=0\right),\\ \; & R_{1}^{T}=\left(\alpha_{1}^{T}=1,\beta_{1}^{T}=1\right),\;R_{1}^{A}=\left(\alpha_{1}^{A}=1,\beta_{1}^{A}=1\right),\;R_{1}^{B}=\left(\alpha_{1}^{B}=0,\beta_{1}^{B}=0\right),\\ \; & R_{2}^{T}=\left(\alpha_{2}^{T}=0,\beta_{2}^{T}=1\right),\;R_{2}^{A}=\left(\alpha_{2}^{A}=1,\beta_{2}^{A}=0\right),\;R_{2}^{B}=\left(\alpha_{2}^{B}=1,\beta_{2}^{B}=1\right),\\ \; & R_{3}^{T}=\left(\alpha_{3}^{T}=1,\beta_{3}^{T}=0\right),\;R_{3}^{A}=\left(\alpha_{3}^{A}=1,\beta_{3}^{A}=1\right),\;R_{3}^{B}=\left(\alpha_{3}^{B}=0,\beta_{3}^{B}=1\right),\\ \; & R_{4}^{T}=\left(\alpha_{4}^{T}=0,\beta_{4}^{T}=0\right),\;R_{4}^{A}=\left(\alpha_{4}^{A}=1,\beta_{4}^{A}=1\right),\;R_{4}^{B}=\left(\alpha_{4}^{B}=1,\beta_{4}^{B}=1\right),\\ \; & R_{5}^{T}=\left(\alpha_{5}^{T}=1,\beta_{5}^{T}=1\right),\;R_{5}^{A}=\left(\alpha_{5}^{A}=0,\beta_{5}^{A}=0\right),\;R_{5}^{B}=\left(\alpha_{5}^{B}=1,\beta_{5}^{B}=1\right),\\ \; & R_{6}^{T}=\left(\alpha_{6}^{T}=1,\beta_{6}^{T}=0\right),\;R_{6}^{A}=\left(\alpha_{6}^{A}=0,\beta_{6}^{A}=1\right),\;R_{6}^{B}=\left(\alpha_{6}^{B}=1,\beta_{6}^{B}=1\right), \end{array}$$

where $R_{i}^{T}=R_{i}^{A}⊕R_{i}^{B}$ (i.e., $\alpha_{i}^{T}=\alpha_{i}^{A}⊕\alpha_{i}^{B}$, $\beta_{i}^{T}=\beta_{i}^{A}⊕\beta_{i}^{B}$), and i=0,1,⋯,6.

**Figure 4:**
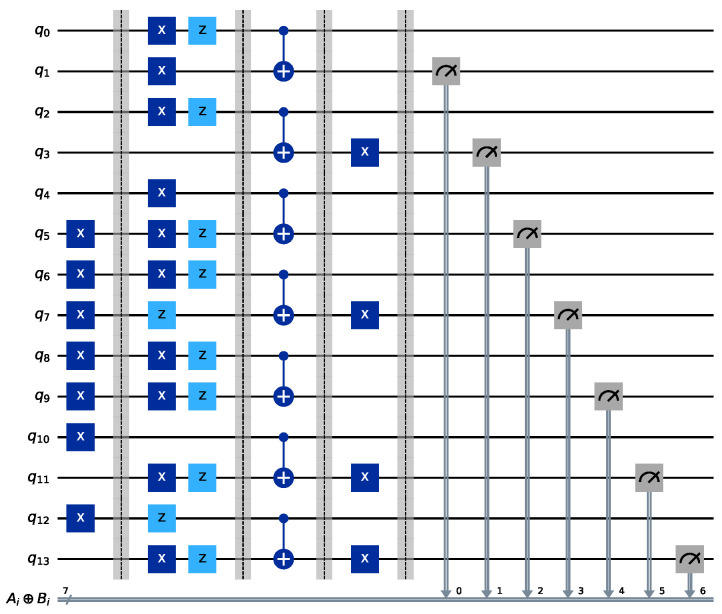
CNOT homomorphic evaluation quantum circuit diagram. In this circuit, registers $q_{0},q_{2},q_{4},q_{6},q_{8},q_{10},q_{12}$ denote the quantum states ($|A_{0}\rangle ,|A_{1}\rangle ,\cdots ,|A_{6}\rangle$) prepared by Alice, while $q_{1},q_{3},q_{5},q_{7},q_{9},q_{11},q_{13}$ denote the quantum states ($|B_{0}\rangle ,|B_{1}\rangle ,\cdots ,|B_{6}\rangle$) prepared by Bob. The entire circuit is divided into five stages, separated by barriers, which correspond to the preparation of quantum states, encryption, CNOT evaluation, decryption, and the final measurement.

Afterwards, the quantum homomorphic encryption stage is performed. Alice and Bob send the encrypted state $x^{\alpha_{i}^{A}}Z^{\beta_{i}^{A}}|A_{i}\rangle$ and $x^{\alpha_{i}^{B}}Z^{\beta_{i}^{B}}|B_{i}\rangle$, respectively, to TP. Then, TP performs CNOT evaluation, and the corresponding quantum circuit is shown in Figure 4. The specific parameter settings for the circuit can be found in Equations (19) and (20).

**Figure 5:**
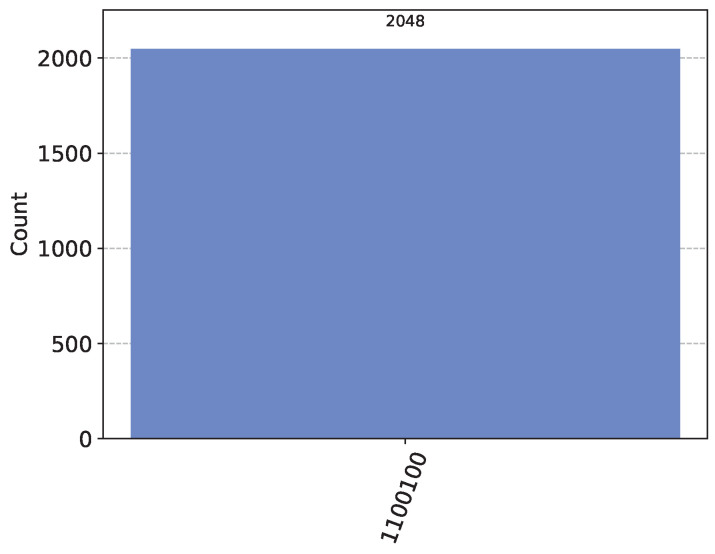
Simulation results of circuits listed in Figure 4. The horizontal axis “11000100” in the figure represents the measurement result of $|A_{i}⊕B_{i}\rangle$, and the result is consistent with the setting of Equation (19). For example, the measurement result of $|A_{6}⊕B_{6}\rangle$ is equal to 1, and the result of $|A_{5}⊕B_{5}\rangle$ is also equal to 1.

The corresponding simulation results are shown in Figure 5. As can be seen from the figure, the measurement results satisfy $A_{i}⊕B_{i}$. The XOR results calculated from the initial states of $|A_{i}\rangle$ and $|B_{i}\rangle$ in Equation (19) are consistent with the simulation results.

In the final privacy set similarity calculation, Alice and Bob first inform TP of the sizes of their private sets, i.e., n=4 and m=3. Then, TP counts the number of measurement outcomes equal to $A_{i}⊕B_{i}=1$ from the homomorphic evaluation, denoted as *l*. From Figure 5, it can be seen that the number of measurements with a result of 1 is 3, i.e., l=3. Based on Equation (7), the sizes of Alice and Bob’s intersection and union are calculated accordingly.

(21) $$\begin{array}{cc} \; & |S_{A}*\cap S_{B}*|=\left(n+m-l\right)/2=\left(4+3-3\right)/2=2\\ \; & |S_{A}*\cup S_{B}*|=n+m-\left(n+m-l\right)/2=4+3-\left(4+3-3\right)/2=5 \end{array}.$$

Thereby, TP can obtain the set similarity of $S_{A}*$ and $S_{B}*$ (i.e., $S_{A}$ and $S_{B}$).

(22) $$\begin{array}{c} J_{AB}=\frac{|S_{A}*\cap S_{B}*|}{|S_{A}*\cup S_{B}*|}=\frac{|S_{A}\cap S_{B}|}{|S_{A}\cup S_{B}|}=\frac{n+m-l}{n+m+l}=\frac{2}{5} \end{array}.$$

This result is the same as directly calculating the similarity between $S_{A}=\left\{2,3,5,6\right\}\subseteq Z_{7}$ and $S_{B}=\left\{1,2,5\right\}\subseteq Z_{7}$. Therefore, the output result of the designed protocol is correct.

### 4.2. Correctness of the Proposed Quantum Privacy-Preserving Range Query Protocol

In this part, we analyze the correctness of the proposed quantum privacy-preserving range query protocol step by step. Assume that Alice has a dataset represented as

(23) $$\begin{array}{cc} \; & x_{1}=2:\left\{d_{1}^{1},d_{1}^{2},\cdots ,d_{1}^{G}\right\}\\ \; & x_{2}=3:\left\{d_{2}^{1},d_{2}^{2},\cdots ,d_{2}^{G}\right\}\\ \; & x_{3}=5:\left\{d_{3}^{1},d_{3}^{2},\cdots ,d_{3}^{G}\right\}\\ \; & x_{4}=6:\left\{d_{4}^{1},d_{4}^{2},\cdots ,d_{4}^{G}\right\} \end{array}.$$

Here, the index numbers $x_{1},x_{2},x_{3},x_{4}$ constitute a privacy set, denoted as SA. Bob’s query range $\left\{y_{1}=1,y_{2}=2,y_{3}=5\right\}$ also forms a set, denoted SB.

Similar to Section 4.1, Alice and Bob first carry out the private set encoding phase, where we also assume k=2. As a result, they obtain the same transformed private sets, i.e., $S_{A}*=\left\{4,6,3,5\right\}$ and $S_{B}*=\left\{2,4,3\right\}$. Correspondingly, the quantum state sequences prepared by Alice and Bob are identical, as shown in Equation (19).

Performing steps 1, 2, and 3, Alice obtains the transformed privacy dataset as follows:

(24) $$\begin{array}{cc} \; & x_{1}^{\prime }=4:\left\{d_{1}^{1}+k_{4}+t_{4},\;d_{1}^{2}+k_{4}+t_{4},\;\cdots ,\;d_{1}^{G}+k_{4}+t_{4}\right\},\\ \; & x_{2}^{\prime }=6:\left\{d_{2}^{1}+k_{6}+t_{6},\;d_{2}^{2}+k_{6}+t_{6},\;\cdots ,\;d_{2}^{G}+k_{6}+t_{6}\right\},\\ \; & x_{3}^{\prime }=3:\left\{d_{3}^{1}+k_{3}+t_{3},\;d_{3}^{2}+k_{3}+t_{3},\;\cdots ,\;d_{3}^{G}+k_{3}+t_{3}\right\},\\ \; & x_{4}^{\prime }=5:\left\{d_{4}^{1}+k_{5}+t_{5},\;d_{4}^{2}+k_{5}+t_{5},\;\cdots ,\;d_{4}^{G}+k_{5}+t_{5}\right\},\\ \; & a_{0}^{\prime }=0:\left\{a_{0}+k_{0}+t_{0},\;a_{0}+k_{0}+t_{0},\;\cdots ,\;a_{0}+k_{0}+t_{0}\right\},\\ \; & a_{1}^{\prime }=1:\left\{a_{1}+k_{1}+t_{1},\;a_{1}+k_{1}+t_{1},\;\cdots ,\;a_{1}+k_{1}+t_{1}\right\},\\ \; & a_{2}^{\prime }=2:\left\{a_{2}+k_{2}+t_{2},\;a_{2}+k_{2}+t_{2},\;\cdots ,\;a_{2}+k_{2}+t_{2}\right\}, \end{array}$$

where $a_{i}$ is randomly generated by Alice, and $k_{i}$ and $t_{i}$ are the keys generated in step 1. $a_{0}^{\prime },a_{1}^{\prime }$, and $a_{2}^{\prime }$ represent the index numbers corresponding to the set that is not part of the set $S_{A}*$.

After completing steps 4, 5, and 6 (i.e., the key generation phase and the quantum homomorphic encryption phase), TP obtains the result of $|A_{i}⊕B_{i}\rangle$. The correctness of this result was already verified in Section 4.1, so it will not be repeated here. TP then focuses on the case where $|A_{i}⊕B_{i}\rangle =|0\rangle$. From the results in Figure 5, it can be concluded that $|A_{0}⊕B_{0}\rangle =|0\rangle$, $|A_{1}⊕B_{1}\rangle =|0\rangle$, $|A_{3}⊕B_{3}\rangle =|0\rangle$, and $|A_{4}⊕B_{4}\rangle =|0\rangle$. TP prepares a new data point $v_{i}$, at the corresponding position, as specified in Equation (16). The specific vector *V* here is $V=[t_{0}-b_{0},t_{1}-b_{1},0,t_{3}-b_{3},t_{4}-b_{4},0,0]$.

After step 7, Alice sends the encrypted private dataset to TP, who then checks whether each set of data is identical, allowing it to infer which positions satisfy $|A_{i}\rangle =|0\rangle$. For the *i*-th set of data, where $|A_{i}⊕B_{i}\rangle =|0\rangle$ and $|A_{i}\rangle =|1\rangle$, TP decrypts it using $v_{i}$ to retrieve a new set of private data. Thus, at the index numbers $x_{1}^{\prime }=4$ and $x_{3}^{\prime }=3$, the private data after TP decryption is

(25) $$\begin{array}{cc} \; & x_{1}^{\prime }=4:\left\{d_{1}^{1}+k_{4}+b_{4},\;d_{1}^{2}+k_{4}+b_{4},\;\cdots ,\;d_{1}^{G}+k_{4}+b_{4}\right\},\\ \; & x_{3}^{\prime }=3:\left\{d_{3}^{1}+k_{3}+b_{3},\;d_{3}^{2}+k_{3}+b_{3},\;\cdots ,\;d_{3}^{G}+k_{3}+b_{3}\right\}. \end{array}$$

For the data corresponding to other index numbers, TP randomly prepares the same data, denoted as $\left\{p_{i}^{1},p_{i}^{2},\cdots ,p_{i}^{G}\right\}$.

Finally, TP sends the data in Equation (25) and $\left\{p_{i}^{1},p_{i}^{2},\cdots ,p_{i}^{G}\right\}$ to Bob. Here, Bob is only concerned with the case where $|B_{i}\rangle =|1\rangle$, because only these positions satisfy $|A_{i}\rangle =|B_{i}\rangle =|1\rangle$ and SA*∩SB*. Combined with the specific assumptions, here, Bob would obtain the data in Equation (25) at positions i=3 and i=4. For other positions *i*, Bob will only obtain the same result $\left\{p_{i}^{1},p_{i}^{2},\cdots ,p_{i}^{G}\right\}$. Thus, for the data $\left\{d_{1}^{1}+k_{4}+b_{4},\;d_{1}^{2}+k_{4}+b_{4},\;\cdots ,\;d_{1}^{G}+k_{4}+b_{4}\right\}$ and $\left\{d_{3}^{1}+k_{3}+b_{3},\;d_{3}^{2}+k_{3}+b_{3},\;\cdots ,\;d_{3}^{G}+k_{3}+b_{3}\right\}$, Bob decrypts using keys $k_{3}$, $k_{4}$ and $b_{3}$, $b_{4}$ to obtain the final query result

(26) $$\begin{array}{cc} \; & \{d_{1}^{1},\;d_{1}^{2},\;\cdots ,\;d_{1}^{G}\},\\ \; & \{d_{3}^{1},\;d_{3}^{2},\;\cdots ,\;d_{3}^{G}\}. \end{array}$$

Based on the initial assumption, Bob’s queried indices are {1,2,5}, while Alice’s set of indices is {2,3,5,6}. Therefore, the result of the query should be the private data corresponding to indices 2 and 5. According to Equation (23), Bob’s query result should be $\left\{d_{1}^{1},\;d_{1}^{2},\;\cdots ,\;d_{1}^{G}\right\}$ and $\left\{d_{3}^{1},\;d_{3}^{2},\;\cdots ,\;d_{3}^{G}\right\}$. Consequently, the output of our protocol is correct.

## 5. Security Analysis

This section will discuss the security of the protocol. In the following, the security of the quantum privacy set similarity comparison protocol is first discussed, followed by analyzing the security of the privacy range query protocol.

### 5.1. Security Analysis for Quantum Privacy Set Similarity Comparison Protocol

For the quantum private set similarity comparison protocol, the security of the protocol primarily depends on the security of the keys generated during the key generation phase, which is closely related to the security of the subsequent homomorphic encryption and CNOT evaluation.

**Theorem 1.**  *With the assistance of a semi-honest quantum cloud TP, Alice and Bob can establish a secure key relationship $R_{j}^{T}=R_{j}^{A}⊕R_{j}^{B}$ using Bell states, ensuring that none of the three parties can access each other’s private information.*

**Proof.**  In the key generation phase, TP randomly prepares Bell states |Φ〉 and |Ψ〉, while Alice and Bob randomly perform measurements in either the Z-basis or the X-basis. Subsequently, Alice and Bob select a subset of particles for eavesdropping detection.

We consider two scenarios: one involving an external eavesdropper, Eve, attempting to steal the key, and the other involving internal eavesdroppers, which include the semi-honest TP as well as dishonest Alice or Bob, trying to obtain the key.

*External attack analysis:* Suppose TP prepares $|\Phi \rangle =\frac{1}{\sqrt{2}}(|00\rangle +\left|11\rangle \right)$ to send to Alice and Bob, and Eve launches an entangle-measure attack [24] on the particles transmitted between TP and Alice. Specifically, Eve utilizes the unitary operation $U_{E}$ to entangle the auxiliary particle, ${|0\rangle }_{E}$, to the target particle and then steals information by observing the state of the auxiliary particle [25]. After Eve’s attack, the state of the system will change to

(27) $$\begin{array}{cc} U_{E}{(|\Phi \rangle |0\rangle }_{E}) & =\frac{1}{\sqrt{2}}(|00\rangle |e_{0}\rangle +|11\rangle |e_{1}\rangle ),\\ \; & =\frac{1}{\sqrt{2}}[\frac{1}{2}(|++\rangle +|-+\rangle +|+-\rangle +|--\rangle |e_{0}\rangle )\\ \; & \;\;+\frac{1}{2}(|++\rangle -|-+\rangle -|+-\rangle +|--\rangle |e_{1}\rangle )] \end{array},$$

where the effect $U_{E}$ can be described as $U_{E}{(|0\rangle |0\rangle }_{E})=|0\rangle |e_{0}\rangle$ and $U_{E}{(|1\rangle |0\rangle }_{E})=|1\rangle |e_{1}\rangle$.

For |Φ〉, when Alice and Bob perform the same measurement basis on the received particles, either in the Z-basis or the X-basis, their measurement results will be identical. This implies that the condition $|e_{0}\rangle =|e_{1}\rangle$ must hold in Equation (27). Only in this scenario can Alice and Bob observe the same results using different measurement bases. Furthermore, satisfying $|e_{0}\rangle =|e_{1}\rangle$ indicates that Eve cannot obtain any secret information from the auxiliary particles. Regardless of the state of the target particle, she will receive the same measurement outcome. Therefore, Eve cannot steal information through the entangle-measure attack without introducing errors.

*Internal attack analysis:* Suppose the semi-honest TP intends to steal Alice’s secret information. In this scenario, since TP plays a role in the initial quantum state preparation, it poses a greater threat than external attackers. TP could prepare false particles, such as single-particle states, to gain access to Alice and Bob’s measurement results. However, this attack cannot evade the eavesdropping detection mechanisms of the protocol. During the eavesdropping detection process, Alice and Bob will verify whether the quantum states prepared by TP meet the required conditions by performing measurements with different bases. Moreover, if TP prepares quantum states |Φ〉 and |Ψ〉 according to the protocol, then TP’s attempt to obtain Alice’s secret information will be the same as an external attacker. Therefore, TP’s attack cannot effectively steal the secret information without introducing an error.

Regarding the case where a dishonest Alice seeks to steal Bob’s key, there is no quantum communication between Alice and Bob during the key generation phase. Therefore, if Alice wishes to steal Bob’s information, she can only attack the particles directly transmitted between TP and Bob. In this scenario, Alice essentially acts as an external attacker; thus, her attack is also ineffective in the absence of errors.

In summary, in the key generation phase, Alice, Bob, and TP can establish a key relationship and cannot obtain each other’s keys. □

In addition, the privacy sets of Alice and Bob are kept confidential. TP can only obtain the size of the privacy sets of Alice and Bob, while Alice and Bob can only know the similarity of the privacy sets.

**Theorem 2.**  *After executing the quantum privacy set similarity comparison protocol, both Alice and Bob’s private sets remain confidential, and TP can only obtain the sizes of Alice and Bob’s private sets.*

**Proof.**  During the privacy set encoding phase of the protocol, Alice and Bob establish a key *k* using the QKD protocol and encode their respective private sets with this key. Without knowledge of the key *k*, external attackers and TP are unable to access the actual private set data. In the homomorphic encryption phase, the quantum states corresponding to the private sets are also encrypted and the transmitted quantum states are all maximally mixed states:

(28) $$\begin{array}{cc} \; & \sum_{\alpha_{i}^{A},\;\beta_{i}^{A}\in \left\{0,1\right\}}X^{\alpha_{i}^{A}}Z^{\beta_{i}^{A}}|A_{i}\rangle \langle A_{i}|X^{\alpha_{i}^{A}}Z^{\beta_{i}^{A}}=\frac{I_{2}}{2},\\ \; & \sum_{\alpha_{i}^{B},\;\beta_{i}^{B}\in \left\{0,1\right\}}X^{\alpha_{i}^{B}}Z^{\beta_{i}^{B}}|B_{i}\rangle \langle B_{i}|X^{\alpha_{i}^{B}}Z^{\beta_{i}^{B}}=\frac{I_{2}}{2}. \end{array}$$

TP as well as the external attacker cannot effectively perform decryption to obtain useful information. Finally, in the set similarity calculation phase, Alice and Bob only inform TP about the size of their respective sets. Therefore, the proposed protocol can ensure the security of Alice and Bob’s private set data without causing information leakage. □

### 5.2. Security Analysis for Quantum Privacy-Preserving Range Query Protocol

In the privacy-preserving range query protocol, the focus needs to be on ensuring the security of Bob’s query range and the corresponding query results, as well as on protecting the security of the data in Alice’s dataset.

**Theorem 3.**  *After executing the privacy-preserving range query protocol, Bob’s query range and query results as well as Alice’s dataset are confidential.*

**Proof.**  In the proposed protocol, Alice’s data consist of two parts: the index numbers of IoT devices and the private data collected by the corresponding devices. Bob’s query range is the IoT device index numbers, and the query content is the data collected by those respective devices.

The protocol considers the IoT device index numbers collected by Alice and the query range of Bob as two private sets and executes the set similarity comparison protocol described in Section 3.1 to determine the query range. Section 4.1 analyzed the security of the set similarity comparison protocol, so the security of the protocol in the query range determination process is guaranteed.

Additionally, for the private data collected by IoT devices, Alice, Bob, and TP establish key relationships through QKD, denoted as $K_{AB}=\left\{k_{0},k_{1}\cdots ,k_{N-1}\right\}$, $K_{AT}=\left\{t_{0},t_{1},\cdots ,t_{N-1}\right\}$, and $K_{BT}=\left\{b_{0},b_{1},\cdots ,b_{N-1}\right\}$, respectively. Alice encrypts her data using keys $K_{AB}$ and $K_{AT}$ before sending it to TP. TP then decrypts the data at the corresponding positions using keys $K_{AT}$ and $K_{BT}$ and forwards the result to Bob. Finally, Bob uses keys $K_{AB}$ and $K_{BT}$ to decrypt and obtain the query results that match the specified query range. During this process, since TP lacks key $K_{AB}$, TP cannot independently decrypt Alice’s private data. For Bob, TP only sends the encrypted data that correspond to the query range. Bob then decrypts these data to obtain the final query results. Therefore, Bob only gains access to the specific query results, without any of Alice’s other data.

Finally, in terms of Bob’s query results and query range, Alice cannot identify Bob’s exact query range since she is unaware of the specific comparison of the index numbers. Furthermore, TP securely transmits the private query results directly to Bob using the quantum secure communication protocol, preventing Alice from obtaining any knowledge of the results. For TP, the encrypted index numbers from both Bob and Alice ensure that TP cannot determine the actual query range. Moreover, without key $K_{AB}$, TP is also unable to retrieve the query results. □

## 6. Discussion

In the proposed quantum privacy-preserving range query protocol designed for IoT environments, three main quantum sub-protocols are included: the QHE-based quantum private set similarity comparison protocol, the quantum key distribution (QKD) protocol, and the quantum secure direct communication (QSDC) protocol. The following analysis will focus on the quantum resource requirements, communication overhead, protocol complexity, and performance.

Firstly, in terms of quantum resource requirements, the proposed set similarity comparison protocol only requires Bell states, single particles, and simple unitary operations combined with projection measurements. As for the other two quantum protocols, they can also be implemented using single particle states and projection measurements [26,27]. Therefore, the overall quantum resource demand of the protocol is not complex, making the proposed quantum privacy-preserving range query protocol feasible under current technological conditions.

Secondly, consider the communication overhead of the protocol. In our protocol, the quantum communication process includes the following steps. (1) Key generation phase: To ensure data security, pairs of participants—Alice and TP, Alice and Bob, and Bob and TP—use a QKD protocol to establish encryption keys for protecting their private data. For instance, in the BB84 protocol, generating an *N*-bit key requires approximately 4N qubits of communication [28]. Thus, the communication overhead for this phase is around 3×4N qubits. (2) Homomorphic encryption phase: During this phase, a series of Bell states are shared between Alice, Bob, and TP to generate keys necessary for homomorphic encryption. This process requires around 8N Bell states to produce *N* keys, resulting in a total communication overhead of approximately 2×8N qubits transmitted between TP, Alice, and Bob. Then, Alice and Bob transmit *N* encrypted quantum states to TP for homomorphic evaluation. (3) Data transmission: After key generation and homomorphic encryption, Alice securely transmits her encrypted quantum states using a QSDC protocol. Then, TP also transmits encrypted quantum states to Bob via a QSDC protocol for final decryption to obtain query results. Typically, a QSDC protocol requires about 4N qubits to securely transmit an *N*-bit of information, such as in the protocol of Deng et al. [29]. Therefore, in order to achieve an *N*-bit privacy range query, the overall quantum communication overhead is approximately 3×4N+2×8N+2N+2×4N=38N qubits.

Thirdly, regarding the protocol’s complexity, in the QKD and QSDC protocols, the two communicating parties need to exchange qubits and use classical communication to share measurement basis information for subsequent measurement operations. This implies that the communication complexity and computational complexity are proportional to the number of qubits *N*, i.e., O(N). For the set similarity comparison protocol, the privacy set encoding stage requires executing a single QKD protocol, which has a complexity of O(N). In the key generation stage, Alice, Bob, and TP establish key relationships using Bell states and single-particle measurements, and this stage also has a complexity of O(N). Finally, in the quantum homomorphic encryption stage, the CNOT evaluations, decryption, and measurement operations performed by TP are proportional to the number of photons *N*. Thus, the complexity of this stage is O(N). Therefore, considering all the above, the overall complexity of the proposed protocol is O(N).

In terms of protocol performance, our protocol leverages the properties of quantum homomorphic encryption to perform set similarity comparison and range queries without revealing the private data of either party. This encryption method allows operations to be conducted directly on encrypted data, thus avoiding the leakage of the original sensitive data. Additionally, the QKD and QSDC protocols securely transmit keys and query results through quantum channels, effectively preventing eavesdropper’s attacks and enhancing the overall security of the protocol.

Furthermore, we conducted a comparison between our proposed quantum privacy-preserving range query scheme and previous related works, as outlined in Table 3. As shown in Table 3, our protocol provides users with long-term security guarantees. With the advancement of quantum technology, previous schemes [8,9,10] based on classical encryption may become vulnerable to attacks from quantum computers, leading to the leakage of private data. Furthermore, our approach leverages quantum homomorphic encryption, ensuring that even a powerful quantum cloud TP cannot extract any secret information from the encrypted quantum states.

In summary, the proposed quantum privacy-preserving range query protocol offers advantages in security and privacy protection. With the application of QHE, data processing and query operations can be performed in the encrypted domain, thereby reducing the risk of exposing the original sensitive information.

## 7. Conclusions

In this work, we proposed a quantum privacy-preserving range query scheme for IoT environments, along with corresponding circuit simulations and performance analysis. The scheme models the data owner’s index and the query user’s range as private sets, with the query range being determined through our quantum private set similarity comparison protocol. The query results are then securely transmitted via QKD and QSDC protocols. In the quantum private set similarity comparison protocol, we first employ a QKD protocol to establish keys and encode private sets into quantum states. Then, quantum homomorphic encryption with CNOT evaluation is used to perform privacy-preserving data comparisons, enabling the comparison of set similarity. Our proposed quantum privacy-preserving range query scheme ensures long-term security with the help of quantum cryptography.

Moreover, our scheme requires only simple and easily prepared quantum states, such as Bell states and single-photon states, as information carriers. Users need only perform basic operations like CNOT, X, and Z gates and projection measurements. This makes the protocol feasible for practical implementation in the IoT ecosystem.

## Figures and Tables

**Table 1:** Results corresponding to different types of Bell states and measurement bases.

Case	Bell State Type $R_{j}^{T}$	Measurement Basis of Alice	Measurement Basis of Bob	Alice’s Results $R_{j}^{A}$	Bob’s Results $R_{j}^{B}$
1	|Φ〉, $R_{j}^{T}=0$	Z-basis	Z-basis	|0〉	|0〉
2	|Φ〉, $R_{j}^{T}=0$	Z-basis	Z-basis	|1〉	|1〉
3	|Φ〉, $R_{j}^{T}=0$	X-basis	X-basis	|+〉	|+〉
4	|Φ〉, $R_{j}^{T}=0$	X-basis	X-basis	|−〉	|−〉
5	|Ψ〉, $R_{j}^{T}=1$	Z-basis	Z-basis	|0〉	|1〉
6	|Ψ〉, $R_{j}^{T}=1$	Z-basis	Z-basis	|1〉	|0〉
7	|Ψ〉, $R_{j}^{T}=1$	X-basis	X-basis	|+〉	|+〉
8	|Ψ〉, $R_{j}^{T}=1$	X-basis	X-basis	|−〉	|−〉

**Table 2:** Measurement results and set relationships.

Alice’ State	Bob’s State	Measurement Results	Set Relationships
$i\notin S_{A}*$, $|A_{i}\rangle =|0\rangle$	$i\notin S_{B}*$, $|B_{i}\rangle =|0\rangle$	$|A_{i}⊕B_{i}\rangle =|0\rangle$	${\left(S_{A}*\cup S_{B}*\right)}^{c}$
$i\in S_{A}*$, $|A_{i}\rangle =|1\rangle$	$i\notin S_{B}*$, $|B_{i}\rangle =|0\rangle$	$|A_{i}⊕B_{i}\rangle =|1\rangle$	$S_{A}*-S_{B}*$
$i\notin S_{A}*$, $|A_{i}\rangle =|0\rangle$	$i\in S_{B}*$, $|B_{i}\rangle =|1\rangle$	$|A_{i}⊕B_{i}\rangle =|1\rangle$	$S_{B}*-S_{A}*$
$i\in S_{A}*$, $|A_{i}\rangle =|1\rangle$	$i\in S_{B}*$, $|B_{i}\rangle =|1\rangle$	$|A_{i}⊕B_{i}\rangle =|0\rangle$	$S_{A}*\cap S_{B}*$

**Table 3:** Comparison between our protocol and previous related works.

Protocols	Method	Security Level	Long-Term Security	Query Range	QHE- Based
Ref. [8]	CHE	CS	No	Privacy	/
Ref. [9]	XOR + hash-based authentication	CS	No	Public	/
Ref. [10]	CHE + PC	CS	No	Public	/
Ref. [16]	QSP + QSMCX + QPQ	QS	Yes	Privacy	No
Ref. [17]	Quantum OSID + QPQ	QS	Yes	Privacy	No
Our protocol	QPSSC + QKD + QSDC	QS	Yes	Privacy	Yes

Note. CHE: classical homomorphic encryption; QHE: quantum homomorphic encryption; PC: privacy comparison; CS: classical security; QS: quantum security; QSP: quantum secret permutating; QSMCX: quantum secure multi-party computing XOR; QPQ: quantum privacy query; OSID: oblivious set inclusion decision; QPSSC: quantum privacy set similarity comparison.

## Data Availability

The original contributions presented in the study are included in the article, further inquiries can be directed to the corresponding author.
